# Development and validation of the social media perception scale for preservice physical education teachers

**DOI:** 10.3389/fpsyg.2023.1179814

**Published:** 2023-05-17

**Authors:** Yue Xu, Fangfei Li, Zhihua Yin, Mingzhu Sun, Zhen Guo, Bo Liu

**Affiliations:** ^1^Department of Physical Education and Sport Sciences, University of Limerick, Limerick, Ireland; ^2^College of Physical Education and Health, East China Normal University, Shanghai, China; ^3^Department of Physical Education and Military Training, Zhejiang A&F University, Hangzhou, China; ^4^Department of Physical Education Teaching, Shanghai University of Engineering Science, Shanghai, China; ^5^Division of Sports Science and Physical Education, Tsinghua University, Beijing, China

**Keywords:** preservice physical education teachers, social media, perception, scale development, exploratory factor analysis, confirmatory factor analysis

## Abstract

**Background:**

Social media has become a mainstay of preservice physical education teachers’ professional development. However, previous studies have been dominated by qualitative research, and there is still a lack of quantitative research based on samples from eastern countries. The objective of this study is to develop and validate of the Social Media Perception Scale for Preservice Physical Education teachers (SMPS-PPE).

**Method:**

Items of questionnaire created from 70 concepts of the perception model described in our previous study. Questionnaire survey was used to collect quantitative data from a sample of 977 preservice physical education teachers through surveys. We analyzed the data using SPSS 26.0 and AMOS 24.0, conducting item analysis, exploratory factor analysis and confirmatory factor analysis to examine the data.

**Results:**

SMPS-PPE consists of 26 items grouped into three factors: value perception, risk perception, and overall perception. Our findings indicate that SMPS-PPE has acceptable content validity, internal structure validity, and internal consistency.

**Conclusion:**

SMPS-PPE is a reliable and valid measurement to evaluate social media perception among preservice physical education teachers. Future studies should include larger and more diverse teacher samples to enhance generalizability. The SMPS-PPE should also be modified to better cater to the specific requirements of school teachers and university-based teacher educators in the field of physical education.

## 1. Introduction

Social media has emerged as a prevalent and widely used form of new media, embraced by youth demographic. As for the youngest cohort of physical education teachers, preservice physical education teachers, who possess an insatiable curiosity but lack teaching guidance and experience, social media presents a double-edged sword of opportunities and challenges. On one hand, social media offers various benefits such as free access to professional knowledge, skills, and opinions, serving as a primary source for news retrieval, and providing avenues for global communication. As a result, social media has become an essential tool for professional development among physical education teachers (Carpenter and Harvey, 2020a). On the other hand, the prevalence of social media has introduced new challenges in managing and processing health-related information in physical education. Filter failure is a significant challenge for preservice physical education teachers who struggle to sort out irrelevant information from social media feeds, resulting in information overload and feelings of being overwhelmed (Hobbs, 1997; Ong and Cabañes, 2018). This, in turn, contributes to technostress and other media disorders such as anxiety, depression, headaches, and eye strain, potentially hindering the professional development of preservice physical education teachers (Ong et al., 2019; Lanuza, 2020; Lanuza and Arguelles, 2022; Ong and Tapsell, 2022). In addition, media disorders such as addiction, misinformation, and echo chambers can be detrimental to individuals and society as a whole (Hobbs, 1997, 2010, 2018). Additionally, the use of manipulative algorithms in social media has led to the proliferation of networked propaganda, sustained by online communities and reinforced through a powerful feedback loop between mainstream media, politicians, and internet subcultures. This networked propaganda is distributed through a wide range of outlets, both controlled by propagandists and independent, and is characterized by its ability to assume the properties of credible sources. Deeply, the use of these algorithms to distribute and receive news has led to a sense of cynicism toward media and has made it challenging to adopt a consistently truth-focused strategy without being expelled from the network and losing influence in relevant segments of the public (Mihailidis and Cohen, 2013; Mihailidis and Foster, 2021). Moreover, the growing prevalence of fake news on social media is a concern, especially in fields such as physical education and health (Goodyear et al., 2019). Hence, the dissemination of false information through social media can harm the reputation and credibility of preservice physical education teachers and have a negative impact on their professional standing. To overcome these challenges, preservice physical education teachers must be aware of the consequences of fake news and use social media responsibly and knowledgeably to ensure effective professional development.

The potential influence of preservice physical education teachers’ perception of social media has on their involvement in social media usage as a professional learning tool in new era. Thus, it is essential for developing and verifying a effective tool of social media perception among preservice physical education teachers. Several studies have explored the perception of social media among physical education teachers to address the issues faced by preservice physical education teachers. Most of these studies have used a qualitative approach. For example, Goodyear et al. (2019) explored the characteristics of #pechat, a Twitter-based professional learning community used by physical education teachers to engage with each other and share practices (Goodyear et al., 2019). The researchers found that #pechat is a representative example of an established group of practitioners who use social media for professional development. McNamara et al. (2021) focused on communication and learning processes in physical education teachers and found that physical education teachers were more likely to engage in communication through learning activities such as exchanging knowledge, networking, posting or viewing motivational content, and learning about employment opportunities (McNamara et al., 2021). Hyndman and Harvey (2019) determined pre-service teachers’ perceptions of the potential of using Twitter within Health and Physical Education Teacher Education training. They found that values such as facilitating learning, technology engagement, capturing international insight, enhancing collaboration and communication, were perceived. However, some barriers such as receiving adequate training, privacy, excessive technology use, and determining Twitter’s functionality and application across educational contexts were identified (Hyndman and Harvey, 2019). Hyndman and Harvey (2020) investigated preservice teachers’ perceptions of the value of using Twitter for health and physical education teacher education. They found that physical education teachers valued autonomy, relatedness, and competence. However, there were also concerns due to Twitter’s public exposure to undesired Twitter users and how to navigate the platform (Hyndman and Harvey, 2020). Carpenter and Harvey (2020b) compared and contrasted the previous findings related to social media usage among physical education teachers. They suggested that current research informs the wise use and non-use of social media for professional development and learning by those involved in the field of physical education and sport pedagogy. The research illustrates the positives, negatives, and tensions associated with social media use for professional development and learning (Carpenter and Harvey, 2020a).

While there has been a considerable amount of research exploring the perception of social media among physical education teachers, little attention has been given to examining this topic within Eastern countries. To address this gap, Xu et al. (2023) conducted research focused on one of the largest Eastern countries with a significant number of physical education teacher education programs (Xu et al., 2023). While their study employed qualitative research methods, guided by the bottom-up grounded theory, it comprehensively explored the social media perception structure of pre-service physical education teachers and identified the basic elements of their social media perception. It should be noted that qualitative research is inherently more subjective than quantitative research and typically relies on a smaller participant pool. Though some studies used quantitative methods via questionnaire survey to investigate the use of social media (Popovic et al., 2021; Viana et al., 2021; Sari et al., 2022), but the objectives of preservice physical education teachers and eastern samples are still under-research. As a result, it is essential to conduct further objective quantitative research to validate and refine a scientific evaluation scale for assessing social media perception among pre-service physical education teachers. This tool would serve as a reliable and practical means of universally evaluating pre-service physical education teachers’ social media perception levels in the future. Moreover, the development and implementation of this tool could enhance the methodology for comprehending and addressing pre-service physical education teachers’ social media perceptions.

To address the lack of validated instruments that can scientifically explain the complexity of social media perception among preservice physical education teachers, this study is to develop and validate the Social Media Perception Scale for Preservice Physical Education Teachers (SMPS-PPE). We created a questionnaire survey based on the previously constructed social media perception model (Xu et al., 2023) and administered it to 977 preservice physical education teachers in China to assess the reliability and validity of the scale. SMPS-PPE developed and described here has the potential to contribute to methodology for understanding and addressing perceptions of social media for preservice physical education teachers engaged with technology change for improvement in future practice.

## 2. Methods

### 2.1. Item generation

The Convergence model (Creswell and Clark, 2017) was employed in this study to analyze both qualitative and quantitative data. By triangulating the findings, a more comprehensive understanding of the research questions was obtained. The qualitative phase of the study, which provided rich data for analysis, has already been published in Xu et al. (2023). The quantitative phase will validate the findings and offer insight into the perception of social media among preservice physical education teachers, as outlined below:

Xu et al. (2023) conceptualized social media perception model for preservice physical education teachers (Xu et al., 2023). It clearly defined 70 concepts that Social Media Perception Scale for Preservice Physical Education teachers (SMPS-PPE) scale is intended to measure. Xu et al. (2023) involved conducting a thorough review of the existing literature, consulting with experts in the field, collecting online posts and conducting semi-structured interviews with preservice physical education teachers. Specifically, Xu’s study utilized a mixed methods approach to gather data from various sources. To begin with, a comprehensive literature search was conducted using both domestic and international databases including CNKI (China National Knowledge Infrastructure) and Web of Science. The objective of this search was to collect information on the perception model of social media among pre-service physical education teachers. A total of 48 relevant articles in Chinese and 203 in English were obtained and analyzed to extract initial concepts and support the theoretical framework proposed in the study. In addition, social media posts related to physical education, such as those under hashtags #pechat and #pe, were gathered from popular social media platforms such as WeChat, Twitter, and TikTok. Finally, semi-structured interviews were conducted with 17 pre-service physical education teachers to gain a deeper understanding of their views and experiences regarding the use of social media in their profession. It suggested that reliability for generating items of SMPS-PPE. Hence, these 70 concepts used for the Pretest version of the SMPS-PPE construction, details can be found in Table 1.

**Table 1:** *t*-test for high and low grouping of each item in SMPS-PPE (Pre-test version) (*N* = 114).

Items	*t*	*p*
1. I find the graphic videos related to sports or teaching practices in social media smooth and clear	3.386	0.001**
2. I think there is a lack of communication boards for issues related to physical education	3.822	0.000**
3. I think pre-service physical education teachers are happy to share or exchange motor knowledge skills in social media	4.560	0.000**
4. I find it difficult to screen the authenticity of basic sports theory or specific sports data in social media	2.507	0.015*
5. I believe that pre-service physical education teachers can demonstrate strong professional ethics in their interactions with users	4.213	0.000**
6. I feel that the information on sports skills practice and sports theory is too fragmented	2.249	0.029*
7. I think that physical education students and teachers are using social media	4.121	0.000**
8. I feel that pre-service physical education teachers who lack teaching experience are prone to misunderstandings or arguments during the sharing process	4.139	0.000**
9. I feel that there is a lack of authoritative sources of information such as frontline physical education teachers, sports research experts and official sports bodies	0.449	0.655
10. I feel that the division between motor skills and sport theory in social media is unclear	2.843	0.006**
11. I think that in the future, all social media will be interconnected to make it easier to access physical education resources	2.853	0.006**
12. I feel that social media resources for physical education widen the only access to knowledge and skills for pre-service physical education teachers	4.088	0.000**
13. I feel that for pre-service physical education teachers the typographic interface of social media is clean and clear	3.588	0.001**
14. I feel that social media has freed pre-service physical education teachers from the constraints of uniform offline teaching content and pace	5.564	0.000**
15. I feel that social media will encourage the sharing of knowledge on sports, health and nutrition, fitness skills in the future	4.217	0.000**
16. I found that pre-service physical education teachers were more proactive in engaging in discussions on physical education topics in an anonymous setting	3.309	0.002**
17. The limited camera angles of live or recorded video may affect the learning of technical movements	4.357	0.000**
18. I think the future of online sports courses in social media will be more systematic and complete	4.516	0.000**
19. I feel that in the future there will be more and more clear categories of knowledge about sports skills practice and sports theory	4.053	0.000**
20. I found the video clips and other applications needed to share motor skills very easy to use	2.568	0.013*
21. I think the video replay and the speed change feature make the dynamics of the techniques and tactics of each event easier to understand	4.319	0.000**
22. I am concerned that my personal information can be easily compromised or stolen	3.990	0.000**
23. I believe that most of the latest sports information and statistics available on social media are true and reliable	3.407	0.001**
24. I feel that the intelligent pushing of sports information meets the needs of pre-service physical education teachers for learning about different sports specialties	5.991	0.000**
25. I think that the vehicles of picture, sound and text are very suitable for the overall development of the professional knowledge of pre-service physical education teachers	4.733	0.000**
26. I feel that social media enables pre-service physical education teachers to communicate across school and classroom boundaries	6.819	0.000**
27. I believe that resources in social media can meet the needs of pre-service physical education teachers at different levels	4.888	0.000**
28. I feel that the language translation function has helped pre-service physical education teachers to connect internationally	4.115	0.000**
29. I think it’s time consuming to find the right sports resources in a social media world where resources are confusingly categorized	2.824	0.007**
30. I feel that social media provides a database of physical education information that pre-service physical education teachers need	5.695	0.000**
31. I believe that sport resources in social media enable pre-service physical education teachers to connect their daily lives with sport	6.597	0.000**
32. I feel that intelligent push content can inspire me about physical education (e.g., health education perspectives)	5.707	0.000**
33. I find that social media makes it easy for me to collaborate and communicate with in-service physical education teachers	5.125	0.000**
34. I feel that there was a lively atmosphere of discussion about motor skills in the virtual community, topic discussions and other divisions	6.710	0.000**
35. I feel that it is easy to retrieve and collect sports information in social media	5.790	0.000**
36. I feel that paid sports resources in social media are affordable	6.669	0.000**
37. I feel that sports videos, graphics, etc. are very suitable for pre-service PE teachers to build sport representations	6.913	0.000**
38. I feel it is difficult to receive timely responses from high level physical education teachers or research experts in social media	4.585	0.000**
39. I feel that social media has made it easy for pre-service PE teachers to collaborate on interdisciplinary communication	4.834	0.000**
40. I am worried that my fellow students and teachers on campus will misunderstand or make fun of what I post on social media	5.206	0.000**
41. I find physical education resources through social media helpful in reinforcing what I have learned in class	5.745	0.000**
42. I feel that most of the technical aspects of physical education related sharing in social media are not clearly presented	4.598	0.000**
43. I think social media can help break down the unequal distribution of resources for physical education	7.246	0.000**
44. I find that there are many users in social media who are interested in exercise and fitness	3.969	0.000**
45. I feel that the different scales of interaction met the different communication needs of pre-service physical education teachers	6.913	0.000**
46. I feel that the effectiveness of learning in social media is vulnerable to offline noise or other distractions	6.490	0.000**
47. I feel that social media lacks a screening mechanism for outdated poor quality and fraudulent sports information	7.048	0.000**
48. I feel that there are many authoritative physical education experts in social media	5.803	0.000**
49. I feel that most sports knowledge skills in social media are not very credible	3.222	0.002**
50. I feel that funny emojis in social media meet the social and entertainment needs of pre-service physical education teachers	3.600	0.001**
51. I feel that the resources in social media can serve pre-service physical education teachers’ understanding and practice of the details of skill movements	5.701	0.000**
52. I am concerned that pre-service physical education teachers do not have the quality of self-discipline to concentrate on learning about physical education in social media	2.110	0.040*
53. I feel that positive physical activity messages can ignite my enthusiasm for extracurricular exercise	7.898	0.000**
54. I feel that most of the active sports users are motivated by the intention of selling skills courses or nutritional supplements, etc.	2.799	0.007**
55. I think the vivid and colorful interface matches the esthetics of all pre-service physical education teachers	4.418	0.000**
56. I feel that social media has integrated the information resources I need for my major	4.554	0.000**
57. I feel that the capabilities of social media can support multiple perspectives on the endless appeal of sport	8.497	0.000**
58. I think social media can provide me with scientific and effective exercise training programs	5.244	0.000**
59. I think pre-service physical education teachers have access to a lot of information content outside the classroom	6.837	0.000**
60. I found conflicting explanations of physical skills training methods in different sports social media	3.698	0.001**
61. I am concerned that asking for unsolicited professional information related to physical education may be perceived as an invasion of privacy	2.497	0.016*
62. I feel that other users in social media are friendly toward pre-service physical education teachers	2.246	0.029*
63. The information on social media about non-sports professions makes me feel a big cultural difference	2.534	0.014*
64. I feel that the size of the online user base of physical education teachers and related practitioners is large	8.749	0.000**
65. I feel that the knowledge and skills acquired are only applicable to non-physical education majors who are physical education enthusiasts	3.756	0.000**
66. I found the exchange of physical education type topics carried out by pre-service physical education teachers to be sincere and in-depth	4.441	0.000**
67. Seeing my classmates follow, bookmark and retweet the key points of physical education makes me feel very stressed	5.377	0.000**
68. I believe that social media has solved the learning and training paradox faced by many pre-service physical education teachers	7.400	0.000**
69. I feel that the novelty of motor skills in social media is more engaging than offline classes	4.797	0.000**
70. I feel that the large number of categories of information makes it difficult to focus on content relevant to the subject	3.755	0.000**

*indicates *p* < 0.05; **indicates *p* < 0.01.

### 2.2. Participants and procedure

As previously mentioned, we developed the Pre-test version of SMPS-PPE, which includes 70 items and six basic information items (gender, ethnicity, geography, grades, type of university, and level of university). The SMPS-PPE was scored using a 5-point Likert scale, which is common in the research of social media perception (Bicen and Uzunboylu, 2013; Caliskan et al., 2018; Yildiz, 2019). All 70 items scored on a five-point Likert scale: 1 = “strongly disagree,” 2 = “disagree,” 3 = “undecided,” 4 = “agree,” 5 = “strongly agree.” The higher the score, the higher the level of social media perception from preservice physical education teachers or the stronger the importance of influencing factors. And the SMPS-PPE was administered through Sojump,^1^ a professional online survey platform widely used in China (Zhang et al., 2017; Mei and Brown, 2018). To ensure the reliability of the returned SMPS-PPE, the system recorded the commit time, completion time, IP address, and city of respondents.

To ensure the face validity of items, we followed a similar procedure as in the initial study (McNamara et al., 2022). From August 2021 through September 2021, We distributed a draft of the items of SMPS-PPE to 78 undergraduate students majoring in physical education at East China Normal University (ECNU) and requested their feedback regarding the items, such as suggestions for minor alterations in wording and sentence structure and checking for redundancy. All 70 items were then reviewed, revised, and compiled based on their feedback.

To test the internal structural validity of SMPS-PPE, we followed the recommendations of Costello and Osborne (2005) and utilized three different samples: one for item analysis, another for exploratory factor analysis (EFA), and a third for confirmatory factor analysis (CFA) (Costello and Osborne, 2005), see below for details:

Firstly, we generated the Pre-test version of SMPS-PPE, consisting of 70 items. From October 2021 through November 2021, we distributed this Pre-test version of SMPS-PPE to a sample of 114 Chinese preservice physical education teachers. This sample used for the item analysis. Table 2 provides further details regarding this sample.

**Table 2:** Information of participants for item analysis by SMPS-PPE (Pre-test version) (*N* = 114).

Variables	Content	Fre.	Percent (%)
Sex	Male	64	56.1
	Female	50	43.9
Ethnicity	Han	81	71.1
	Minority	33	28.9
Location	Eastern	34	29.8
	Middle	37	32.5
	Western	24	21.1
	North-Eastern	19	16.6
Grade	First	29	25.5
	Second	20	17.5
	Third	38	33.3
	Fourth	27	23.7
Type of university	Comprehensive University	44	38.6
	Normal College	29	25.4
	Sports College	41	36.0
Level of university	985	19	16.7
	211	22	19.3
	Key	34	29.8
	Average	39	34.2

(1) According to the Classification Methods of Eastern, Central and Western Regions and Northeast Region released by the National Bureau of Statistics, the eastern region refers to Beijing, Tianjin, Hebei, Shanghai, Jiangsu, Zhejiang, Fujian, Shandong, Guangdong, and Hainan; the central region refers to Shanxi, Anhui, Jiangxi, Henan, Hubei, and Hunan; the western region refers to Inner Mongolia, Guangxi, Chongqing, Sichuan, Guizhou, Yunnan, Tibet, Shaanxi, Gansu, Qinghai, Ningxia, and Xinjiang; and the northeast region refers to Liaoning, Jilin, and Heilongjiang; (2) Universities in 985 and 211 are highly selective and competitive, and their graduates are highly sought after by employers in China and around the world. For the rank, 985 is better than 211, 211 is better than key, and the average. The same explanations as the tables of participants’ information below.

Next, we created a Beta version of the SMPS-PPE based on the data analysis of the Pre-test version. Different samples were collected to perform exploratory factor analysis (EFA). Participants were recruited using the same procedures described above, and the samples were collected at two different time periods using different internet links. From December 2021 through January 2022, we then distributed this Beta version of the SMPS-PPE to a sample of 429 Chinese preservice physical education teachers, as shown in Table 3.

**Table 3:** Information of participants for exploratory factor analysis (EFA) by SMPS-PPE (Beta version) (*N* = 429).

Variables	Content	Fre.	Percent (%)
Sex	Male	287	66.9
	Female	142	33.1
Ethnicity	Han	314	73.2
	Minority	115	26.8
Location	Eastern	145	33.8
	Middle	115	26.8
	Western	69	16.1
	North-Eastern	100	23.3
Grade	First	113	26.4
	Second	126	29.4
	Third	141	32.9
	Fourth	49	11.3
Type of university	Comprehensive University	147	34.2
	Normal College	123	28.7
	Sports College	159	37.1
Level of university	985	114	26.6
	211	94	21.9
	Key	102	23.8
	Average	119	27.7

Finally, we administered the final version of the SMPS-PPE, consisting of 26 items. Similarly, from February 2022 through March 2022, we then distributed this final version of the SMPS-PPE to a sample of 434 Chinese preservice physical education teachers. The samples were collected to perform confirmatory factor analysis (CFA). Participants were recruited using the same procedures described above, and the samples were collected at different time periods using different internet links. Please refer to Table 4 for further details regarding this sample.

**Table 4:** Information of participants for confirmatory factor analysis (CFA) by SMPS-PPE (Final version) (*N* = 434).

Variables	Content	Fre.	Percent (%)
Sex	Male	288	66.4
	Female	146	33.6
Ethnicity	Han	244	56.2
	Minority	190	43.8
Location	Eastern	172	39.6
	Middle	127	29.3
	Western	34	7.8
	North-Eastern	101	23.3
Grade	First	54	12.5
	Second	126	29.0
	Third	113	26.0
	Fourth	141	32.5
Type of university	Comprehensive University	158	36.4
	Normal College	129	29.7
	Sports College	147	33.9
Level of university	985	109	25.1
	211	107	24.6
	Key	104	24.0
	Average	114	26.3

### 2.3. Measures and statistical analyze

In the first stage, to assess the appropriateness, feasibility, and relevance of the scale items to the intended constructs, we performed an item analysis on the first sample (*N* = 114). We utilized the Critical Ratio method to determine the deletion of items based on the achievement of the critical value, along with homogeneity tests and correlation analysis. Since the SMPS-PPE was scored on a five-point Likert scale, it is standard practice to use the Critical Ratio method and correlation analysis to conduct item analysis (Bryant and Yarnold, 1995).

In the second stage, to statistically determine the number of factors and items that should be retained in the SMPS-PPE, we conducted a series of EFAs on the second sample (*N* = 429). We used KMO value Bartlett’s test and varimax rotation, as recommended for scale development by Kaiser (1974). Following Tabachnick and Fidell (2001), the KMO value 0.60 and higher value of KMO test is essential for good factor analysis (Tabachnick and Fidell, 2001). We then conducted a factor analysis to determine the number of factors (Matsunaga, 2010; Wu, 2010; Escobar-Viera et al., 2018), with the following criteria: (i) each item has a commonality of >0.40; (ii) each factor contained at least three items; (iii) items were consistent with factor meanings and difficult to combine with other items.

In the third stage, CFA has been recommended over the use of exploratory factor analysis as it provides a more rigorous method of examining construct validity by enabling comparisons of alternative *a priori* theoretical models (Kline, 1998). Before CFA, using the third sample (*N* = 434), we assessed the reliability and validity of the final version of the SMPS-PPE. Subsequently, we tested the three factors and the structural equation model of the SMPS-PPE that emerged from the EFA analyses through CFA using AMOS version 24.0 software. To determine an acceptable model fit, we assessed the following criteria: GFI, AGFI, IFI, NFI, TLI, and CFI above 0.90, while RMSEA <0.1 (Hu and Bentler, 1999; Schermelleh-Engel et al., 2003; Kline, 2013).

## 3. Results

### 3.1. Item analysis

Critical Ratio Method. Using independent sample *t*-tests, the items of SMPS-PPE were divided into high (top 27% of total score) and low (bottom 27% of total score) groups to examine their relationship with the total score of the SMPS-PPE (Fan, 1954). Based on the criteria for significant differences, items that did not meet the criteria were removed. Items 4, 6, 9, 20, 52, 61, 62, and 63 were found to not meet the criteria, while other items had significant discrimination (*p* < 0.01). Please refer to Table 1 for further details. These results indicated that the remaining items in the pre-test version of SMPS-PPE had high discrimination and better discriminatory power.

Correlation analysis method. The correlation analysis method is used to explore the relationship between the total score of SMPS-PPE and the scores of each item in SMPS-PPE. Under the precise guidance of the correlation coefficient standard, items with correlation coefficients that do not meet the standard should be deleted. Specifically, Based on Ebel’s (1951) recommendations for developing scales, items with a discriminant index below 0.20 should be eliminated (Ebel, 1951). To assess the relationship between each item and the overall scores of SMPS-PPE, a Pearson correlation analysis was conducted separately for each of the 114 Pre-test version of SMPS-PPE returned. The correlations between each of the 70 items and the overall scores of the SMPS-PPE were highly significant (*p* < 0.01, see Table 5 for details), with correlation coefficients greater than 0.40. This indicates that each item was highly related to the overall SMPS-PPE and was closely related to the psychological trait or underlying behavior being measured.

**Table 5:** Correlation between the 70 items and total score of SMPS-PPE (Pre-test version) (*N* = 114).

Number	Average	Standard deviation	Correlation coefficient	Number	Average	Standard deviation	Correlation coefficient
1	3.880	1.0943	0.619^**^	37	3.880	0.9351	0.813^**^
2	3.300	1.2102	0.467^**^	38	3.780	0.9907	0.553^**^
3	3.820	1.1315	0.627^**^	39	3.660	0.9125	0.659^**^
5	3.700	0.9692	0.613^**^	40	3.180	1.2979	0.534^**^
7	4.100	0.9482	0.596^**^	41	3.980	0.9533	0.713^**^
8	3.400	1.1721	0.458^**^	42	3.380	1.0228	0.680^**^
12	3.820	1.0767	0.535^**^	43	3.600	0.9640	0.783^**^
13	3.600	0.9211	0.607^**^	44	4.000	0.9640	0.617^**^
14	3.480	1.0869	0.576^**^	45	3.840	0.9505	0.759^**^
15	4.100	0.8103	0.703^**^	46	3.660	1.1479	0.639^**^
16	3.700	1.0299	0.506^**^	47	3.800	1.1371	0.634^**^
17	3.900	1.0101	0.627^**^	48	3.420	1.0841	0.592^**^
18	3.980	1.0539	0.684^**^	49	3.280	1.0644	0.429^**^
19	4.060	0.9081	0.637^**^	50	3.500	1.1055	0.652^**^
21	4.000	0.8989	0.570^**^	51	3.680	0.9732	0.655^**^
22	3.120	1.1658	0.416^**^	53	3.920	0.9394	0.723^**^
23	3.400	0.9428	0.603^**^	55	3.540	1.0093	0.616^**^
24	3.560	0.9673	0.677^**^	56	3.240	1.1817	0.634^**^
25	3.980	0.9099	0.723^**^	57	4.000	0.8040	0.721^**^
26	3.940	0.9301	0.783^**^	58	3.540	1.0291	0.584^**^
27	3.560	0.9673	0.656^**^	59	3.860	0.8764	0.709^**^
28	3.880	0.9773	0.690^**^	60	3.540	0.9684	0.427^**^
30	3.520	1.1054	0.699^**^	64	3.480	0.9043	0.614^**^
31	3.680	0.9307	0.736^**^	65	3.120	1.0943	0.380^**^
32	3.740	1.0012	0.749^**^	66	3.680	0.6799	0.532^**^
33	3.840	0.9070	0.730^**^	67	3.240	1.0162	0.510^**^
34	3.660	1.0562	0.663^**^	68	3.480	0.8100	0.566^**^
35	3.740	0.8718	0.764^**^	69	3.380	0.8502	0.520^**^
36	3.200	1.3027	0.640^**^	70	3.640	0.8935	0.390^**^

*indicates *p* < 0.05; **indicates *p* < 0.01.

Based on the results of both Critical Ratio Method and Correlation Analysis Method, items 4, 6, 9, 20, 52, 61, 62, and 63 removed from the SMPS-PPE. Therefore, a total of 62 items were deemed suitable and retained used for developing and validating SMPS-PPE continuingly.

### 3.2. Exploratory factor analysis

A questionnaire named SMPS-PPE (Beta Version) with the remaining 62 items was created for EFA. In the process of EFA, we conducted KMO and Bartlett’s tests, maximum variance, and factor naming. More information on the analysis can be found below:

#### 3.2.1. KMO and Bartlett’s tests

To assess the suitability of the SMPS-PPE for EFA, the KMO and Bartlett’s tests were conducted on the total of 62 items from SMPS-PPE (Beta Version). The coefficient analysis showed a KMO value of 0.904 and a highly statistically significant (*p* < 0.01) *χ*^2^ value of 1,667.535 for the Bartlett’s spherical test (refer to Table 6 for details). The KMO value of 0.904 indicates that the correlation between the items is suitable for factor analysis since it is not significantly different. Additionally, the Bartlett’s spherical test results rejected the null hypothesis, indicating that the individual items of SMPS-PPE are not independent of each other, and there are common factors present between the correlation matrices of the parent groups, making them suitable for factor analysis. These results provide consistent information that supports the suitability of the SMPS-PPE for EFA (Table 7).

**Table 6:** KMO and Bartlett’s sphericity test results for the SMPS-PPE (Beta version) (*N* = 429).

KMO values	Bartlett ‘s test	
0.904	*χ*^2^ (approximate cardinality)	1,667.535
	df (degrees of freedom)	153
	Sig. (*p*)	0.000**

*indicates *p* < 0.05; **indicates *p* < 0.01.

**Table 7:** Variance explained results for the SMPS-PPE (Beta version) (*N* = 429).

Factor number	Characteristic roots			Explanation of variance before rotation			Explanation of variance after rotation		
	Characteristic roots	Explanation of variance%	Cumulative%	Characteristic roots	Explanation of variance %	Cumulative%	Characteristic roots	Explanation of variance%	Cumulative%
1	13.979	53.765	53.765	13.979	53.765	53.765	7.154	27.515	27.515
2	2.431	9.351	63.116	2.431	9.351	63.116	6.443	24.780	52.294
3	1.854	7.131	70.247	1.854	7.131	70.247	4.668	17.953	70.247
4	0.945	3.636	73.883	–	–	–	–	–	–
5	0.852	3.277	77.160	–	–	–	–	–	–
6	0.691	2.659	79.819	–	–	–	–	–	–
7	0.617	2.371	82.190	–	–	–	–	–	–
8	0.538	2.071	84.262	–	–	–	–	–	–
9	0.485	1.867	86.128	–	–	–	–	–	–
10	0.458	1.762	87.890	–	–	–	–	–	–
11	0.373	1.434	89.324	–	–	–	–	–	–
12	0.347	1.334	90.658	–	–	–	–	–	–
13	0.319	1.225	91.883	–	–	–	–	–	–
14	0.310	1.193	93.076	–	–	–	–	–	–
15	0.273	1.049	94.125	–	–	–	–	–	–
16	0.235	0.902	95.027	–	–	–	–	–	–
17	0.220	0.845	95.872	–	–	–	–	–	–
18	0.183	0.702	96.574	–	–	–	–	–	–
19	0.171	0.657	97.231	–	–	–	–	–	–
20	0.153	0.590	97.821	–	–	–	–	–	–
21	0.148	0.569	98.391	–	–	–	–	–	–
22	0.115	0.443	98.834	–	–	–	–	–	–
23	0.104	0.399	99.232	–	–	–	–	–	–
24	0.089	0.341	99.573	–	–	–	–	–	–
25	0.058	0.222	99.795	–	–	–	–	–	–
26	0.053	0.205	100.000	–	–	–	–	–	–

#### 3.2.2. Maximum variance

After conducting a principal component analysis with Varimax rotation on the initial 62 items of the SMPS-PPE (Beta Version), 36 components were retained based on the Kaiser-Guttman rule (Kaiser, 1960). Items 2, 3, 4, 7, 9, 11, 12, 13, 18, 19, 20, 21, 23, 25, 26, 32, 33, 36, 37, 38, 40, 43, 44, 46, 48, 49, 51, 53, 55, 56, 57, 58, 59, 60, 61, and 62 were removed based on a threshold of <0.4 for the loading coefficients. The remaining 26 items met the requirements and are presented in Table 8. The factor loadings ranged from 0.648 to 0.817, and all items in SMPS-PPE had a commonality of >0.40. Factor 1 (10 items) included items 1, 5, 8, 10, 14, 27, 35, 45, 50, and 54. Factor 2 (9 items) included items 15, 16, 17, 24, 28, 29, 31, 42, and 47. Factor 3 (7 items) included items 6, 22, 30, 34, 39, 41, and 52.

**Table 8:** Results of the correlation matrix for the SMPS-PPE (Beta version) (*N* = 429).

	Overall perception	Value perception	Risk perception	Total score
Overall perception	1.000	0.705^**^	0.761^**^	0.923^**^
Value perception	0.705^**^	1.000	0.773^**^	0.881^**^
Risk perception	0.761^**^	0.773^**^	1.000	0.895^**^
Total scale	0.923^**^	0.881^**^	0.895^**^	1.000

*indicates *p* < 0.05; **indicates *p* < 0.01.

**Figure 1:**
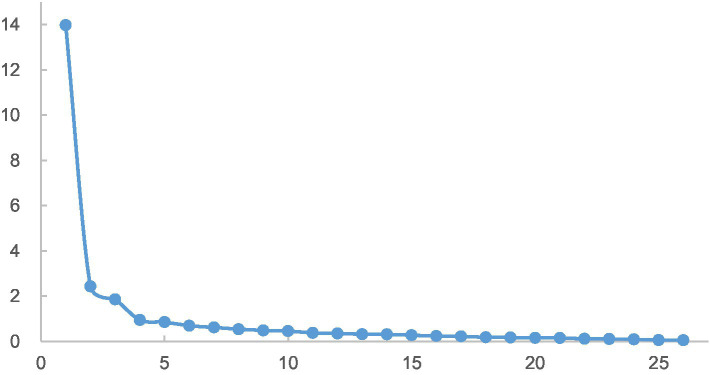
Gravel map.

Furthermore, the steep slope plot (see Figure 1) shows that the slope tends to flatten out after the third principal component, indicating that retaining three factors is appropriate.

#### 3.2.3. Factor naming

After analyzing the items of SMPS-PPE, we found that all items were aligned with the professional expectations in the field of physical education. There was a strong correspondence between factors and items, and we named factor 1 as Overall Perception, factor 2 as Value Perception, and factor 3 as Risk Perception. After completing the EFA, we deleted items 2, 3, 4, 7, 9, 11, 12, 13, 18, 19, 20, 21, 23, 25, 26, 32, 33, 36, 37, 38, 40, 43, 44, 46, 48, 49, 51, 53, 55, 56, 57, 58, 59, 60, 61, and 62. Totally, 44 items were removed from the SMPS-PPE of pre-test version (Table 9).

**Table 9:** Rotation matrix of three factor loadings of 26 items in the SMPS-PPE (Beta version) (*N* = 429).

Item		Public factor		
		Factor 1	Factor 2	Factor 3
A50.	I found that the size of the online user base related to physical education is very large	0.817	0.321	0.224
A5.	I find myself surrounded by students and teachers who use social media a lot	0.796	0.243	0.124
A1.	I felt that the sport or teaching related graphics and videos were presented smoothly and clearly	0.793	0.324	0.194
A8.	I feel that for pre-service PE teachers social media typography is simple and easy to understand	0.753	0.326	0.186
A45.	I think the color scheme and other designs are in line with the aesthetics of pre-service PE teachers	0.737	0.141	0.250
A27.	I feel that sports information in social media is easy to retrieve and collect and organize	0.719	0.361	0.246
A35.	I think social media can help break down the unequal distribution of resources for physical education	0.704	0.434	0.121
A54.	I feel that the content shared by users in social media is fragmented	0.703	0.427	0.209
A10.	I find that information content such as sports and fitness has a high level of interest in social media	0.668	0.431	0.107
A14.	I feel that in the future there will be a clearer classification of content related to the teaching of physical education	0.664	0.476	0.140
A16.	I think social media can provide me with cutting-edge information on physical education	0.272	0.778	0.307
A17.	I feel that social media integrates the learning of physical education majors	0.369	0.767	0.151
A31.	I think an inclusive sharing atmosphere is good for pre-service PE teachers to develop interdisciplinary perspectives	0.313	0.765	0.197
A47.	I think the sharing function of social media can showcase all aspects of sport	0.259	0.762	0.185
A29.	I feel that formats such as graphic videos are good for building the image of movement for pre-service PE teachers	0.247	0.724	0.329
A42.	I think funny emojis can meet the social entertainment needs of pre-service PE teachers	0.368	0.704	0.259
A15.	I feel that applications such as replay shifting make the dynamics of technique and tactics more clear	0.402	0.676	0.138
A28.	I feel that access to physical education related information is mostly free or low cost	0.442	0.674	0.193
A24.	I feel that the Smart Push service has enabled me to learn about other knowledge skills outside of sport	0.237	0.648	0.380
A34.	I am concerned that the limited perspective of formats such as video or graphics may affect the learning of technical essentials	0.166	0.262	0.813
A22.	I am concerned that other users may steal or misappropriate my personal knowledge and skills in physical education	0.029	0.196	0.771
A52.	Seeing students around me share information about pre-service PE teachers made me feel heavy pressure	0.063	0.406	0.732
A6.	Pre-service PE teachers who lack teaching experience are prone to misunderstandings or arguments in sharing	0.491	−0.013	0.715
A41.	I feel that most of the sports knowledge and skills shared by users in social media are not very credible	0.185	0.286	0.712
A39.	I think social media lacks a screening mechanism for outdated, poor quality, fraudulent sports information	0.420	0.236	0.697
A30.	I feel it is difficult to receive timely responses from high level PE teachers or experts in social media	0.501	0.123	0.675

### 3.3. Scale structure exploration based on confirmatory factor analysis

Before conducting CFA for SMPS-PPE’s structure exploration, the data from the third round of SMPS-PPE (*N* = 429) were analyzed for reliability, validity:

#### 3.3.1. Reliability analysis

The Cronbach’s coefficient alpha was used to examine the internal consistency reliability of the SMPS-PPE. Table 10 shows that the overall alpha coefficient of the SMPS-PPE was 0.982. The alpha coefficients for the Overall Perception, Value Perception, and Risk Perception factors were 0.907, 0.929, and 0.852, respectively. These results indicate that the consistency or homogeneity among all the items of SMPS-PPE was good, and the scale had high reliability.

**Table 10:** Three factors and alpha coefficients of SMPS-PPE (*N* = 429).

Factors	Overall perception	Value perception	Risk perception	SMPS-PPE
Internal consistency reliability (α coefficient)	0.907	0.929	0.852	0.982

#### 3.3.2. Folded half reliability

After eliminating the required items, the 26 remaining items were sorted into two parts, and the results are displayed in Table 11. The first half had a Cronbach’s alpha coefficient value of 0.948, and the second half had a value of 0.946, both of which exceeded 0.8. Additionally, the correlation coefficient value between the first and second halves was 0.881, and the coefficient of confidence in the fold was 0.936, both of which were also greater than 0.8, indicating high-quality reliability. According to Cronbach’s (1951) criteria, a fold-half reliability greater than 0.8 indicates high-quality reliability (Cronbach and Meehl, 1955). Therefore, all scales met the criteria and can be used in subsequent analyses. To further investigate the consistency of individual items within the SMPS-PPE across dimensions, Table 11 provides the corresponding item-wise statistics for the three dimensions.

**Table 11:** Results of discounted half confidence analysis (*N* = 429).

Cronbach’s alpha coefficient				Correlation coefficient values	Discount factor		Guttman coefficient
First half		Second half		0.881	Equal length	Unequal length	0.937
Value	Number of items	Value	Number of items				
0.948	13	0.946	13		0.937	0.937	

In accordance with psychometric requirements, a Cronbach’s α coefficient greater than 0.9 indicates good reliability, while a split-half reliability greater than 0.8 indicates high-quality reliability. Based on these criteria, this scale meets the standards and can be used for further analysis. To further examine the consistency of each item within the internal dimensions of the SMPS-PPE, Table 12 provides the corresponding item-total statistics for the three dimensions.

**Table 12:** Overall statistics for SMPS-PPE items for each dimension (*N* = 429).

Dimensionality	Title item	Dimensions when items are deleted average	When an item is deleted the scale variance of	Amended total project relevance	Relevance Square	Cronbach at the time of item deletion alpha factor
Overall perception	A50	35.020	57.527	0.866	0.790	0.945
	A5	35.110	56.772	0.824	0.741	0.946
	A1	35.045	57.119	0.831	0.709	0.946
	A8	35.055	57.409	0.845	0.779	0.945
	A45	35.280	57.569	0.693	0.547	0.952
	A27	35.115	56.997	0.829	0.742	0.946
	A35	35.140	57.890	0.676	0.556	0.953
	A54	35.105	57.431	0.857	0.756	0.945
	A10	35.225	58.145	0.798	0.699	0.947
	A14	35.175	58.045	0.782	0.689	0.948
Value perception	A16	31.295	38.822	0.856	0.768	0.948
	A17	31.335	38.736	0.850	0.752	0.948
	A31	31.365	39.198	0.844	0.733	0.949
	A47	31.280	39.479	0.777	0.651	0.952
	A29	31.435	38.579	0.813	0.680	0.950
	A42	31.320	38.792	0.812	0.687	0.950
	A15	31.345	40.157	0.769	0.609	0.952
	A28	31.255	39.528	0.841	0.757	0.949
	A24	31.370	39.129	0.807	0.685	0.951
Risk perception	A34	23.930	22.839	0.879	0.803	0.927
	A22	23.910	23.771	0.836	0.748	0.931
	A52	23.930	24.166	0.747	0.613	0.939
	A6	23.830	24.574	0.764	0.640	0.937
	A41	23.910	23.801	0.820	0.748	0.932
	A39	23.895	24.295	0.744	0.630	0.939
	A30	23.935	23.217	0.875	0.800	0.927

#### 3.3.3. Validity analysis

Various types of scale validity tests exist, such as structural validity, content validity, and calibration validity. However, given that the SMPS-PPE was still in its early stages of development, there was a lack of comparable scale both domestically and internationally. As a result, this study primarily focused on testing content validity and construct validity.

#### 3.3.4. Content validity

The development of this scale involved a systematic process, which included an extensive literature review, online posts collected in social media, personal interviews, and continuous expert consultation (Khan, 2017; Xu et al., 2023). Firstly, a literature review on preservice physical education teachers’ perceptions of social media was conducted both domestically and internationally, including a total of 48 Chinese and 203 English articles were obtained and analyzed to extract preliminary concepts and support the theoretical framework developed in this study. Secondly, 17 preservice physical education teachers were interviewed using semi-structured interviews, and the generated theory underwent a rigorous coding process based on grounded theory. The concepts from this systematic theory were used as a foundation for developing items. Additionally, online posts related to physical education, such as those using the hashtags #pechat and #pe, were collected from popular social media platforms such as WeChat, Twitter, and TikTok. Finally, five professors in physical education were invited to provide guidance on the items, and with the joint efforts of the experts and research assistants, the scale was continually revised and adjusted through several rounds of feedback from test results. Therefore, the scale can be considered to have good content validity.

#### 3.3.5. Structural validity

The correlation coefficients between the three dimensions and the total SMPS-PPE score were found to be 0.923, 0.991, and 0.895, respectively. According to Tuker’s theory (1949), a reasonable level of construct validity is indicated by a correlation between items and the total SMPS-PPE score between 0.30 and 0.80, and a correlation between factors between 0.10 and 0.60 (Tucker, 1951). Therefore, the observed correlation coefficients suggest a reasonable degree of independence between the factors within the SMPS-PPE, and that each factor is measuring what the overall SMPS-PPE intends to measure. Hence, the SMPS-PPE demonstrates good structural validity, as presented in Table 8.

#### 3.3.6. Confirmatory factor analysis

The construction of an ideal theoretical model should take the following criteria into account: (i) The range of variation for GFI, AGFI, NFI, TLI, CFI, and IFI should be between 0 and 1, with values closer to 1 indicating a better fit. (ii) A lower value of *χ*^2^, with *χ*^2^/df ≤ 5 (if 1 < *χ*^2^/df < 2, then the fit is considered good). (iii) Lower values of RMSEA, with RMSEA ≤0.05 (RMSEA ≤0.08 is also acceptable). Furthermore, a good model fit is indicated when *χ*^2^/df < 2, RMSEA <0.05, and the values for GFI, AGFI, NFI, TLI, CFI, and IFI are all above 0.90 (values above 0.80 are also acceptable).

#### 3.3.7. Structural equation model

**Figure 2:**
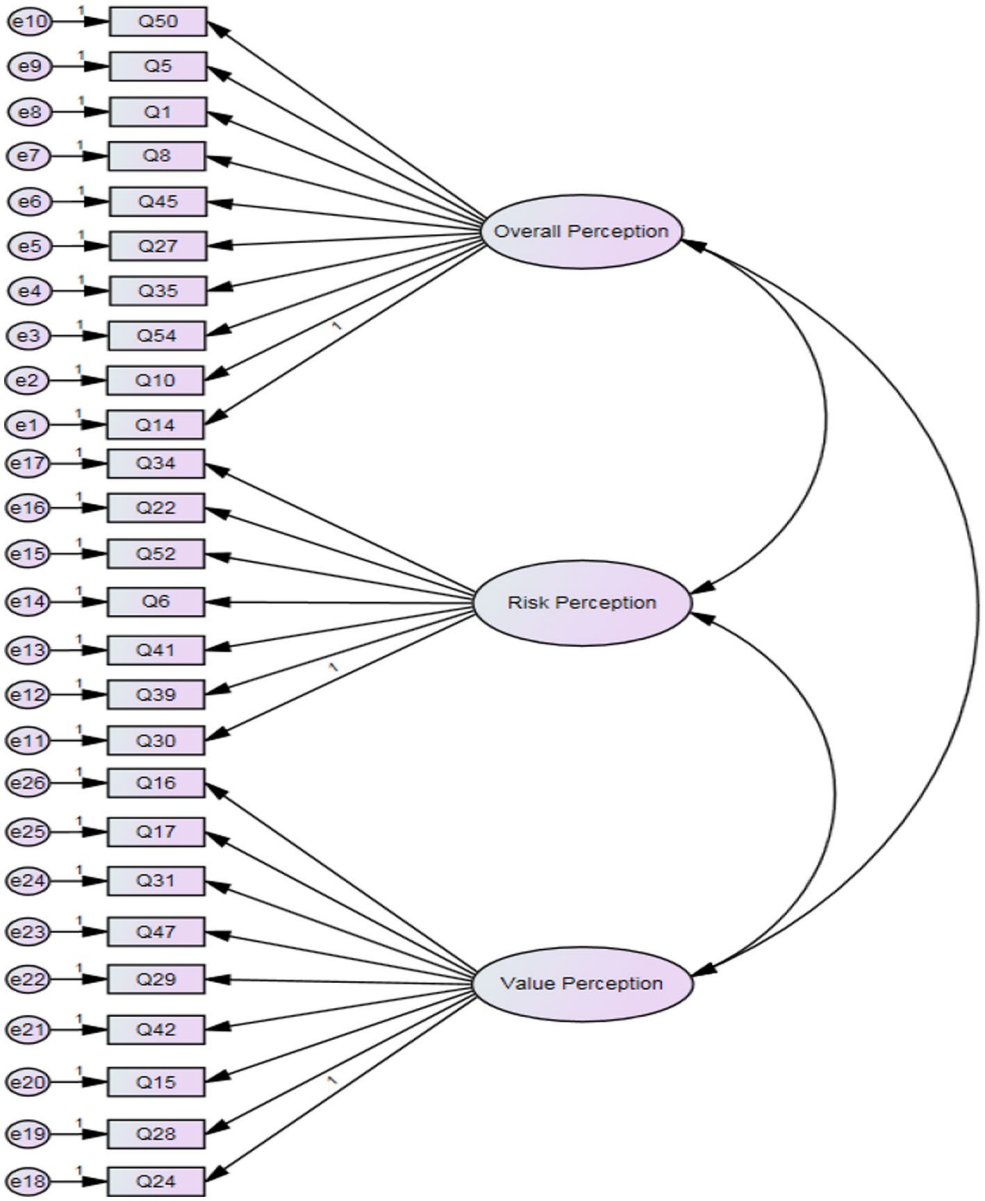
A three-factor model of the SMPS-PPE. e residual error.

This process aimed to validate the scientific nature and model fit of the theoretical model. To achieve this, the covariance matrix of the sample was obtained and used to validate the model. The validation factor analysis results are presented in Table 13, and the structural model plot is shown in Figure 2. The results in Table 13 indicate a good overall fit of the theoretical model for the scale, with *χ*^2^/df = 1.053, GFI = 0.940, RMSEA = 0.012, AGFI = 0.928, CFI = 0.940, IFI = 0.984, and TLI = 0.982.

**Table 13:** CFA model fit results (*N* = 434).

Commonly used standards	*χ*^2^/df	GFI	RMSEA	AGFI	CFI	IFI	TLI
Judgment criteria	<3	>0.9	< 0.1	>0.9	>0.9	>0.9	>0.9
Value	1.053	0.940	0.012	0.928	0.940	0.984	0.982

Upon reviewing Table 13, it can be observed that all the fit indices of the model have achieved the level of good model fit. Therefore, the formal survey data have validated that the three-factor structure is reasonable and valid. The three-factor structure model is illustrated in Figure 2.

## 4. Discussion

This study followed a rigorous procedure for psychological scale development to create the SMPS-PPE. The SMPS-PPE consists of three dimensions, including total of 26 items: overall perception, value perception, and risk perception. Ten items in the overall perception dimension, 9 items in the value perception dimension, and 7 items in the risk perception dimension. The development process included item analysis, EFA, reliability testing and CFA, ensuring that the scale is standardized and rigorous. In this study, we utilized the Convergence model (Creswell and Clark, 2017) to examine the qualitative and quantitative data. By combining the findings, we gained a more comprehensive understanding of the research questions. Our previous publication, Xu et al. (2023), focused on the qualitative phase of the study, which yielded rich data for analysis. In this article, we will present the results of the quantitative phase, which validates the earlier findings and offers new insights into the perceptions of social media among preservice physical education teachers. Specifically, the model constructed in Xu et al. (2023) also found there are three dimensions (Xu et al., 2023), it implies that our studies was consistent with previous studies and theory.

The main purpose of the item analysis was to assess the differentiation between the items in the SMPS-PPE, to prevent redundancy and ensure that each item focused on distinct elements. To ensure accuracy, both the critical ratio method and correlation analysis were used for cross-validation. Specifically, the critical ratio method involved conducting an independent samples *t*-test for 27% of the high and 27% of the low subgroups. After testing for differentiation and correlation, it was found that items 4, 6, 9, 20, 52, 61, 62, and 63 did not meet the criteria, while the remaining 62 test items were highly differentiated (*p* < 0.01), indicating good discriminatory power. Correlation analysis showed that each item in the SMPS-PPE had a correlation greater than 0.40 with the total score of the SMPS-PPE, indicating high homogeneity. Combining the results of the critical ratio method and correlation analysis, it was found that the items in the pre-test version of the SMPS-PPE were highly independent but had a high correlation with the total score of the SMPS-PPE, thus confirming the appropriateness of the items. Then, based on the 429 returned questionnaires, EFA was conducted on the 62-item SMPS-PPE (beta version). After applying the principal component analysis with maximum variance (Varimax) for positive cross-rotation, 36 components were obtained. However, items 2, 3, 4, 7, 9, 11, 12, 13, 18, 19, 20, 21, 23, 25, 26, 32, 33, 36, 37, 38, 40, 43, 44, 46, 48, 49, 51, 53, 55, 56, 57, 58, 59, 60, 61, and 62 did not meet the requirements and were removed, leaving a total of 26 items with loadings that met the criteria. Additionally, we analyzed the reliability and validity of the three dimensions and 26 items in SMPS-PPE. The alpha coefficient of each of the three dimensions and the alpha coefficients of the front and back parts of the scale were all greater than 0.90, indicating that the items in the SMPS-PPE had good consistency and homogeneity, and the SMPS-PPE had high reliability. The validity analysis showed that the content validity of the SMPS-PPE was appropriate, and the correlation matrix coefficients between the items and within the dimensions met the requirements, indicating that the SMPS-PPE was well-structured and had good validity.

Based on the systematic and scientific quantitative analysis process described above, the SMPS-PPE (Official Version) was developed, consisting of 26 items. The SMPS-PPE (Official Version) was administered to 434 participants and subjected to CFA using structural equation modeling. The fit indices of the three-factor structural model, including *χ*^2^/df, GFI, AGFI, IFI, NFI, TLI, CFI, RMR, and RMSEA, all reached the level of a good model fit. This indicates that the three-factor structure of pre-service physical education teachers’ social media perception was found to be reasonable and valid by EFA and CFA. Therefore, the structure of the SMPS-PPE is reasonable and can be validated. Overall, this study culminated in the development of a validated SMPS-PPE.

The SMPS-PPE implicates that social media perception is a complex interrelationship of discourse. Posits that social media perception is a complex interplay of discourse, involving the interrelationships among social media perception, value perception, and risk perception. This intricate relationship can significantly influence the attitudes and behaviors of preservice physical education teachers toward social media. It is crucial for preservice teachers to comprehend that their engagement and perception of social media are shaped by their overall perception of it, as well as their appraisal of its value and associated risks. For instance, preservice physical education teachers can utilize this knowledge to deliberately reflect on their use of social media, taking into account its potential impact on their professional development and instructional practices. By acknowledging the multifaceted and complex nature of these factors, preservice teachers can make informed decisions about how to leverage social media to enhance their knowledge and skills, and keep abreast of the latest advancements in their field.

For the explanation of each dimension, we found that the value dimension of social media is mainly about authenticity and credibility, while digital divide and fragmentation are widely discussed in the risk perception of social media. In social media, authenticity refers to the extent to which social media content reflects the genuine thoughts and behaviors of its creators (Hobbs, 1997, 2018), while credibility refers to the degree to which social media content is perceived as accurate and trustworthy by its consumers (Hobbs, 2010). Based on our data, the use of social media in physical education has both positive and negative impacts on authenticity and credibility. On the positive side, the large online user base and prevalence of social media use among students and teachers create a convenient platform for sharing and organizing sports-related information. The graphics and videos presented on social media are also clear and esthetically pleasing, and the typography is simple and easy to understand. Social media can help break down the unequal distribution of resources for physical education and provide cutting-edge information to pre-service physical education teachers. However, there are also concerns about the authenticity and credibility of information shared on social media. The content shared by users is often fragmented and lacks a screening mechanism for outdated or fraudulent information. Pre-service PE teachers without teaching experience may be prone to misunderstandings or arguments, and the pressure to share credible information can be overwhelming. It is also difficult to receive timely responses from high-level physical education teachers or experts. Overall, while social media can be a valuable tool for sharing information and breaking down barriers, it is important to approach it with caution and critical thinking among preservice physical education teachers.

To summarize, a valid scale for assessing the SMPS-PPE was developed through exploratory and confirmatory factor analysis. The overall perception factor comprises 10 items (1, 5, 8, 10, 14, 27, 35, 45, 50, 54), the value perception factor includes 9 items (15, 16, 17, 24, 28, 29, 31, 42, 47), and the risk perception factor comprises 7 items (6, 22, 30, 34, 39, 41, 52). The scale demonstrated good reliability, indicating that it is a scientifically sound and dependable instrument.

## 5. Conclusion

The social media perception assessment scale developed in this study can serve as a valuable tool for evaluating the current stages of social media perception among preservice physical education teachers. It can also be used to identify current issues reflected in their perception of social media and provide guidance for administrative departments to design education programs for physical education teachers and assess the information technology capabilities of preservice physical education teachers.

## 6. Limitations and future research

As no research is impeccable, the present research of developing and validating SMPS-PPE also possessed some limitations that offer further research opportunities:

To facilitate the professional development of preservice physical education teachers in social media context, it is advisable to implement appropriate assessment tools to measure their social media literacy levels in various regions. For getting a global and comprehensive understanding, this is particularly important in Asian countries where previous research has identified a gap in this area, such as China, India, Korea, and Japan. This will enable preservice physical education teachers to gain a scientific, objective, and comprehensive understanding of their social media perception and provide a reference for improving the application of social media to promote their professional development effectively.

Given the limited sample size and locations of this study, it may not be representative of all preservice physical education across the world, which may affect the generalizability of our results. Thus, it is recommended that future research includes a larger sample size and wide sample locations to further validate and refine the scale, enhancing its applicability and scientific accuracy. Furthermore, in order to ensure that the SMPS-PPE is a valid and reliable assessment tool for measuring social media literacy levels among preservice physical education teachers, it is important to tailor it to the specific needs of this target population. Given that school teachers and university-based teacher educators in physical education have unique requirements, it is recommended that the SMPS-PPE be revised to address these needs. This may involve modifying the language used in the scale, adjusting the format of the questions, or including additional items that are relevant to the specific context of physical education. By doing so, the SMPS-PPE can provide a more accurate assessment of social media literacy levels among this population, which can inform the development of effective strategies for supporting their professional development in this area. Also, another avenue for future research could be to examine the moderating effects of additional demographic factors, such as the frequency of social media use and preferences for specific social media platforms. By doing so, a more comprehensive understanding of social media perception among physical education teachers can be attained, and effective strategies for promoting professional development in this field can be formulated.

## Data availability statement

The raw data supporting the conclusions of this article will be made available by the authors, without undue reservation.

## Ethics statement

The studies involving human participants were reviewed and approved by East China Normal university. The patients/participants provided their written informed consent to participate in this study. Written informed consent was obtained from the individual(s) for the publication of any potentially identifiable images or data included in this article.

## Author contributions

YX, ZY, and FL: conceptualization. ZY, ZG, and BL: methodology. YX: software. YX, MS, and ZY: formal analysis. YX, FL, and ZY: writing—original draft preparation. YX, ZY, MS, and FL: writing—review and editing. ZY, ZG, and BL: funding acquisition. All authors have read and agreed to the published version of the manuscript.

## Funding

This research was funded by the Later funded projects of the China National Social Science Foundation, grant number 21FTYB006; Key Projects of the China National Social Science Foundation, grant number 20ATY009; Sports and Health Project of China Education Society, grant number 2020TY093117ZB; and China Postdoctoral Science Foundation, grant number of 2021M701932.

## Conflict of interest

The authors declare that the research was conducted in the absence of any commercial or financial relationships that could be construed as a potential conflict of interest.

## Publisher’s note

All claims expressed in this article are solely those of the authors and do not necessarily represent those of their affiliated organizations, or those of the publisher, the editors and the reviewers. Any product that may be evaluated in this article, or claim that may be made by its manufacturer, is not guaranteed or endorsed by the publisher.

## Footnotes

^1^ www.wjx.cn
